# A rare case of idiopathic intrauterine intestinal volvulus complicated with intestinal perforation: a case report from Syria

**DOI:** 10.1093/jscr/rjab192

**Published:** 2021-05-27

**Authors:** Victor Khoury, Ammar Omran, Norma Taishori

**Affiliations:** Pediatric Surgery Department, Tishreen University Hospital, Latakia, Syria; Pediatric Surgery Department, Tishreen University Hospital, Latakia, Syria; Pediatric Department, Tishreen University Hospital, Latakia, Syria

## Abstract

Fetal intestinal volvulus is rare, but it is a serious condition due to its life-threatening complications.

The bowel loop becomes twisted; thus, impaired venous return leads to bowel necrosis.

Prenatal volvulus is most secondary to intestinal atresia, arterial supply defect or without any underlying cause, with consideration that cystic fibrosis is the cause of the intestinal obstruction, because of meconium ileus.

We report a case of prenatal volvulus complicated with intestinal perforation and meconium peritonitis in the context of meconium ileus.

## INTRODUCTION

Fetal volvulus is a rare, yet life-threatening condition, which requires skillful diagnosis and management; it occurs when bowel loops become twisted around the mesenteric artery or its branches [1]; prenatally, it is mainly caused by intestinal malrotation or obstruction [2].

Meconium ileus is the earliest clinical manifestation of cystic fibrosis (CF) and the presenting feature in 15–20% of CF newborns [3].

Few cases of surviving infants—particularly cases with meconium peritonitis—have been reported in the literature, and half of the reported cases either died *in utero* or were aborted [4].

The diagnosis can be challenging, as the antenatal clinical presentation is nonspecific [1, 5].

## CASE PRESENTATION

A 2700 g full-term boy was delivered via elective cesarean section, with an uncomplicated pregnancy; Apgar scores were 8 and 9 at 1 and 5 min; neonate examination showed a very tense distended abdomen with signs of peritoneal irritation.

The infant was kept fasted and under surveillance at neonate intensive care unit (ICU) for further investigation; an abdominal X-ray showed dilated small bowel loops with few air-fluid levels. Ultrasonography showed a distended bowel with hyperactivity movement, and laboratory finding showed very high levels of white blood cells with left shift and elevated platelets count.

**Figure 1:**
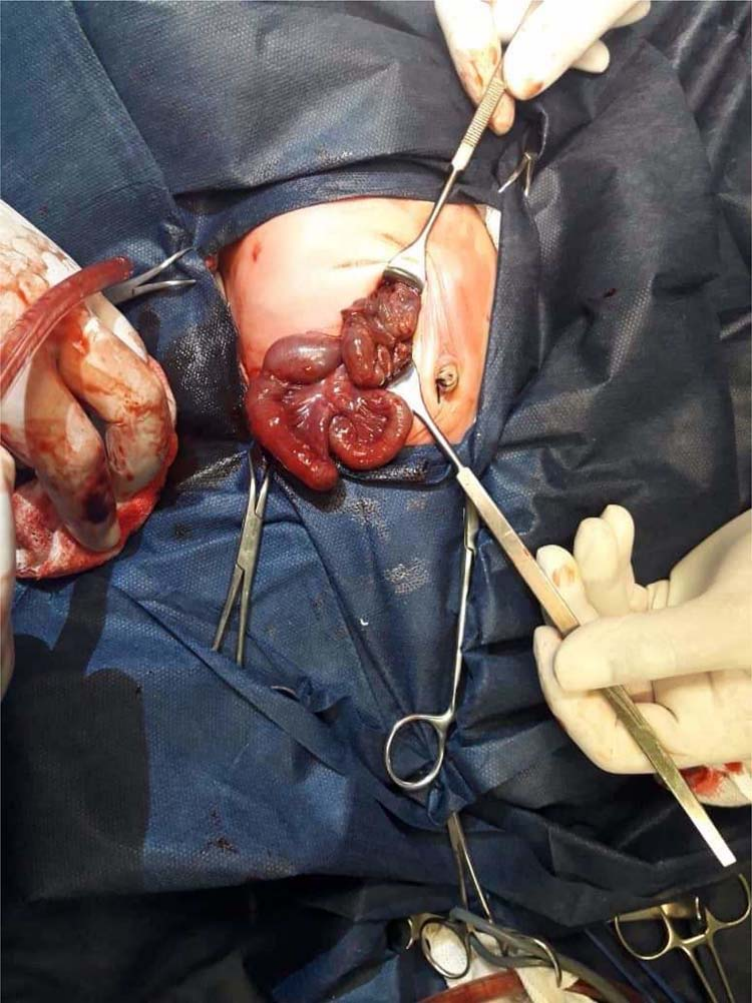
Operative view: ileal segmental volvulus.

**Figure 2:**
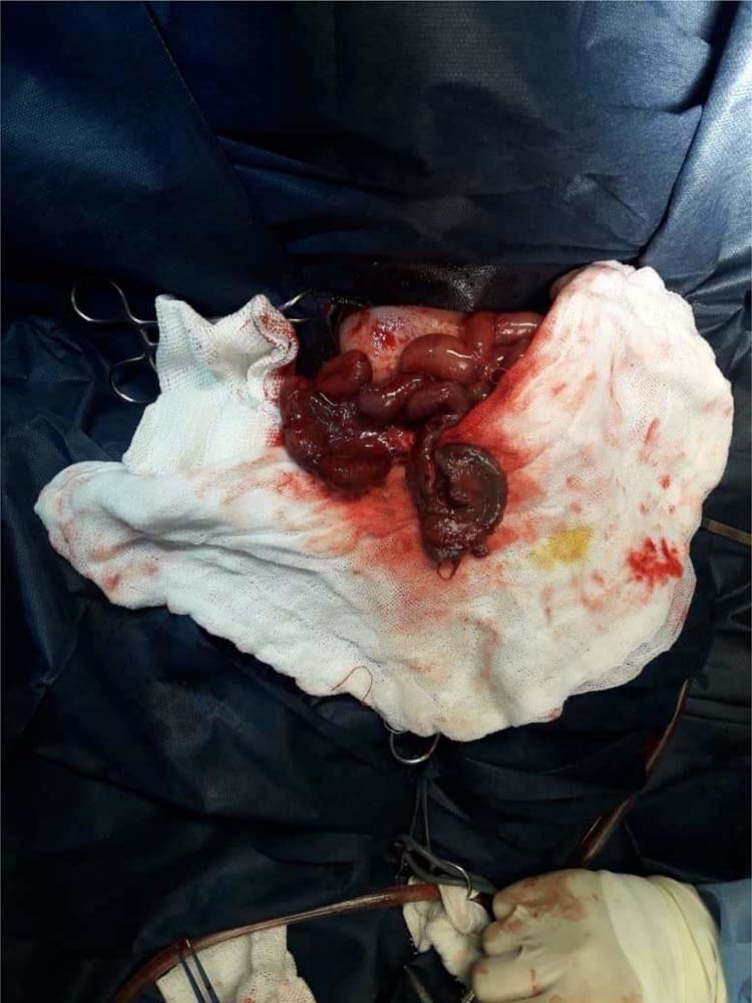
Operative view: necrotic perforated intestinal segment.

A nasogastric tube was inserted but a surgical decision was made when the respiratory effort worsened with an increased in abdominal diameter. An emergent laparotomy found volvulus of the segment of the ileum 30 cm proximal to the ileocecal junction (Fig. 1); this segment was twisted and perforated (Fig. 2), thus was resected with an ileostomy.

**Figure 3:**
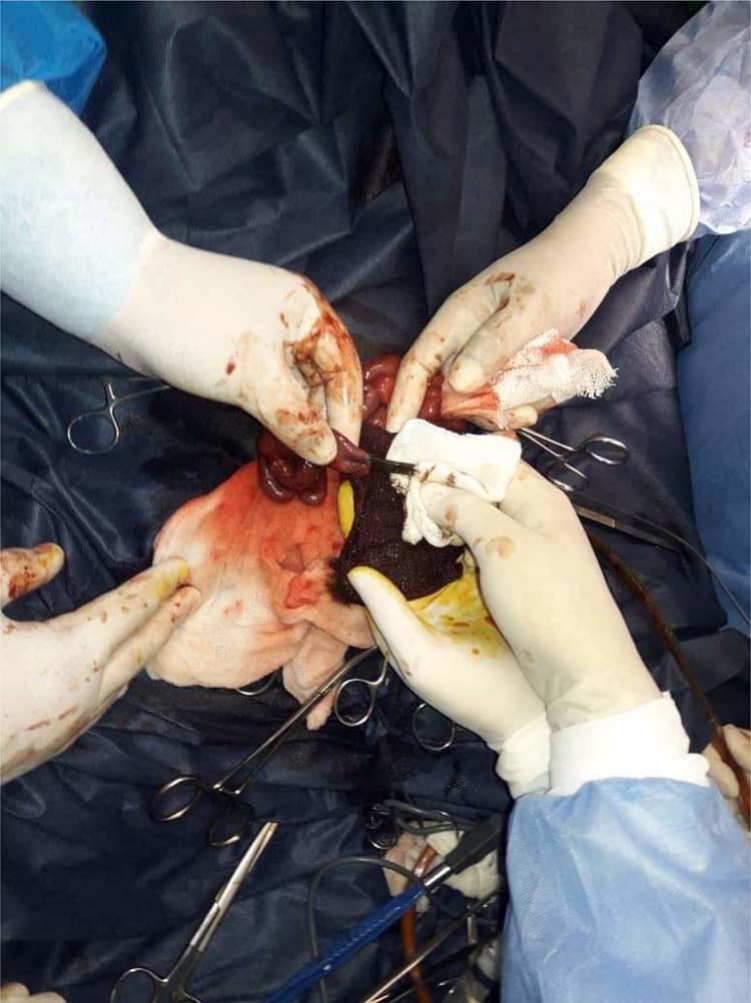
Operative view: viscous thick meconium; being removed by intraluminal irrigation.

During the abdomen cavity explorations, very thick meconium was found (Fig. 3), which rose the suspicion of CF, as the cause of the meconium ileus. The thick meconium was removed by intraluminal irrigation, as long fibrin fibers were found.

After surgery, the baby was admitted to the ICU incubator, with a nothing by mouth diet, broad-spectrum antibiotics and total parental nutrition.

On postoperative (post-op) Day 3, the stoma motility became viable, and we started feeding with milk, which gradually elevated to reach the full daily need on post-op Day 12.

On Day 13, the baby was sent home with good blood results and good weight gain.

The polymerase chain reaction result test for CF was negative but only for the 34 mutations available at our facility’s laboratory.

## DISCUSSION

There are three types of volvulus presenting in the prenatal or newborn period: classic type, segmental type and volvulus without malposition type. Segmental volvulus has been described as twisting of bowel loop, due to an anomaly, such as: meconium ileus (related to CF), atresia, mesenteric defects, duplication mesenteric cyst, congenital diaphragmatic hernia and abdominal wall defects or can be classified as idiopathic. Volvulus without malrotation is an extremely rare surgical condition and is a diagnosis of exclusion [1, 4, 6, 7], with meconium ileus being as the earliest clinical manifestation of CF, occurring into to 20% of patients with CF; that is why CF should be considered when fetal intestinal volvulus has been suspected with impaction of thick and tenacious meconium [2, 6]; in our case the volvulus was idiopathic with no malposition of the intestinal and CF testing were negative.

Presenting features of intrauterine intestinal volvulus may include ultrasonographic signs of fetal intestinal obstruction and perforation (polyhydroaminos, intestinal dilation, a cystic and/or solid abdominal mass, peritoneal calcification and ascites) and general signs of fetal distress (reduced fetal movement, diminished variability oligohydramnios) [4, 8, 9].

The prenatal diagnosis of volvulus without malrotation was advanced when ultrasound scan revealed typical specific signs like whirlpool or snail sign with sensitivity and specificity of 89% and 92%, also, nonspecific signs like cystic mass, dilated bowel loops, peritoneal calcification, ascites and polyhydroaminos.

Despite the improvement in prenatal screening, it’s quite difficult and few cases have been reported in the literature, where a definitive prenatal diagnosis of volvulus was made [2, 4, 6, 7, 10], and that is what we experienced where the ultrasound signs were not specific to the diagnosis of intestinal volvulus and gave us a little indication to distended bowel loops only.

After birth, a tense, distended abdomen, abdominal mass, a dark discoloration of the abdominal wall (Cullen’s sign), bile emesis, failure to pass meconium, bloody diarrhea (in severe cases) and shock are the symptoms most indicative of volvulus, with abdominal X-ray, ultrasound and CT are the diagnostic procedures [4].

In our case, there were no ultrasound signs suggested to volvulus or intestinal perforation and even after birth there were none; thus, the surgical dissection was only made upon the clinical presentation and physical examination and the need to laparotomy based on the primary diagnosis of ‘acute abdomen’.

The indications of surgical intervention include: pneumoperitoneum, intestinal obstruction, progressive peritonitis, sepsis and fixed intestinal loop on plain abdominal film, with various surgical possibilities to treat complicated forms, including: resection with enterostomy, primary anastomosis or purse-string enterotomy, but primary anastomosis is preferred over the stoma, to maximize small bowel length, with the consideration that the long-term prognosis depends on: the level of the intestinal obstruction, on the length of bowel segment involved, on the presence of meconium peritonitis and on the gestational age at birth [1, 6, 10].

We preferred the stoma over primary anastomosis, because of the small weight birth, thus to eliminate the surgery duration, and the presence of intestinal perforation with meconium peritonitis, which lead us to decide that it is safer to avoid the primary anastomosis in this inflammatory condition.

In conclusion, this is an unusual case of prenatal intestinal volvulus complicated with intestinal perforation, due to lacking of diagnostic signs neither in prenatal period, nor in postnatal period, and all the decisions were made upon the surgeon’s instinct of approaching an acute abdomen in a moderate pediatric facility. The baby is doing well and prepared to close the stoma.

## CONFLICT OF INTEREST STATEMENT

None declared.
